# Models for Gut-Mediated Horizontal Gene Transfer by Bacterial Plasmid Conjugation

**DOI:** 10.3389/fmicb.2022.891548

**Published:** 2022-06-30

**Authors:** Logan C. Ott, Melha Mellata

**Affiliations:** ^1^Department of Food Science and Human Nutrition, Iowa State University, Ames, IA, United States; ^2^Interdepartmental Microbiology Graduate Program, Iowa State University, Ames, IA, United States

**Keywords:** horizontal gene transfer, plasmids, bacterial evolution, *in vivo*, *in vitro*, antimicrobial resistance

## Abstract

The emergence of new antimicrobial resistant and virulent bacterial strains may pose a threat to human and animal health. Bacterial plasmid conjugation is a significant contributor to rapid microbial evolutions that results in the emergence and spread of antimicrobial resistance (AR). The gut of animals is believed to be a potent reservoir for the spread of AR and virulence genes through the horizontal exchange of mobile genetic elements such as plasmids. The study of the plasmid transfer process in the complex gut environment is limited due to the confounding factors that affect colonization, persistence, and plasmid conjugation. Furthermore, study of plasmid transfer in the gut of humans is limited to observational studies, leading to the need to identify alternate models that provide insight into the factors regulating conjugation in the gut. This review discusses key studies on the current models for *in silico*, *in vitro*, and *in vivo* modeling of bacterial conjugation, and their ability to reflect the gut of animals. We particularly emphasize the use of computational and *in vitro* models that may approximate aspects of the gut, as well as animal models that represent *in vivo* conditions to a greater extent. Directions on future research studies in the field are provided.

## Graphical abstract

Graphical Abstract Models for gut-mediated bacterial conjugation and plasmid transfer. Depiction of conjugative elements (Left, Top), current *in silico* models (Left, Middle), experimental *in vitro* models (Left, Bottom), and *in vivo* animal models (Right) for bacterial conjugation in the gut. Arthropods; spring tails (*Folsomia candida*), fleas (*Alphitobius diaperinus*), fruit flies (*Drosophila melanogaster*), house flies (*Musca domestica*), beetles (*Xenopsylla cheopis*); *Rhabditidae*; nematodes (*Caenorhabditis elegans*); *Phasianidae*; chickens (*Gallus gallus*). *Leporidae*; rabbits (*Oryctolagus cuniculus*). *Muridae*; mice (*Mus musculus*), rats (*Mus rattus*).

**Figure 1:**
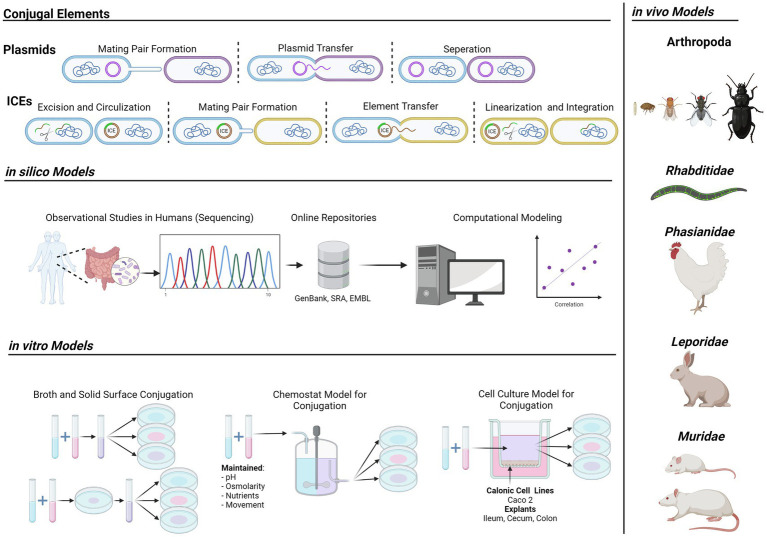
Graphical Abstract

## Introduction

The emergence and spread of antimicrobial resistance (AR) and virulence genes (VG) are of critical significance across the globe (Smillie et al., 2010; Devanga Ragupathi et al., 2019). Bacteria transmit genetic information either vertically from origin cell to progeny cells, or horizontally in the form of conjugation, transformation, or transduction (Smith, 2012; Juhas, 2015). Bacterial conjugation has shown to be the most significant route of horizontal gene transfer (HGT) in the context of the spread and emergence of AR in bacteria (Dolejska and Papagiannitsis, 2018). HGT is prevalent in every environment, such as in soil, on surfaces, in clinical environments, as well as in the gut of animals (Guiyoule et al., 2001; Klümper et al., 2015; von Wintersdorff et al., 2016). HGT is a more significant means of evolution than that of random mutation, which results in slow alterations of genes already in a host bacterium. HGT, instead, results in the acquisition of entire genes, allowing for the rapid expression of new virulence, metabolic, and resistance phenotypes (Javadi et al., 2017). While bacterial evolution has been studied through the process of HGT, the primary concern in the context of human and animal health has been in AR, virulence, and the transfer of such from resistant bacterial strains to previously naïve bacteria that may function as reservoirs for transmission to other naive strains with the potential to become clinically and agriculturally important (Guiyoule et al., 2001; Klümper et al., 2015; von Wintersdorff et al., 2016).

The gut microbiota plays a critical role as a reservoir for AR genes (ARG) and HGT (McAllister et al., 1996; Salyers et al., 2004; Penders et al., 2013; Rolain, 2013). In mammals, as well as poultry, these reservoirs serve as sources for the emergence of novel AR strains with significance in both human and animal health (Balis et al., 1996; Salyers et al., 2004; Penders et al., 2013; Rolain, 2013; Nesme et al., 2014; Chen et al., 2016). However, to our knowledge, there are only few studies into the specific interactions of bacteria carrying one or more mobile plasmids and the host physiology resulting in HGT in humans. This is due to the inherent difficulty of conducting causal experiments in the human gastrointestinal tracts, and relies on either observational studies, or the use of *in vitro* and *in vivo* animal models to represent the human gut.

Many of the factors involved in the host–microbe interactions of gut-mediated HGT are not yet understood (Stecher et al., 2012; Ott et al., 2020). Limited *in vivo* studies have been conducted to determine the roles of factors such as host immunity, antibiotic treatment status, gut environmental conditions, and secretory molecules in the interactions of microbial HGT (Klimuszko et al., 1989; Thomas and Nielsen, 2005; Diaz et al., 2012; Stecher et al., 2012; Huddleston, 2014; Machado and Sommer, 2014; Getino et al., 2015; Zeng and Lin, 2017; Devanga Ragupathi et al., 2019; Ott et al., 2020). While there have been many publications reporting the transfer of AR plasmids *in vitro*, often in the context of soil or wastewater, there has been a stark lack of experiments that demonstrate the interactions of these bacteria *in vivo* (Macuch et al., 1967; Rang et al., 1996; Gevers et al., 2003; Card et al., 2017; Benz et al., 2020). In this review, we will present the current models for *in vitro*, *in silico*, and *in vivo* HGT experimentation regarding the gut environment, as well as survey of important findings demonstrated in these models.

## Conjugative Elements

Classically, there are two forms of conjugative vectors: integrative and conjugative elements, and conjugative plasmids. They both facilitate the transfer of genetic information between two bacterial cells but vary in the intracellular mechanism and maintenance within the cell. Regardless of genetic system, or mobile element involved, there are minimum requirement for both the physical interaction, recognition of recipient cells, and transfer of mobile DNA by donor and recipient cells. This is often mediated through the expression of type four secretion systems (T4SS) such as *tra*, *trw*, and *trb*. Each of these genes encode either intra or extracellular proteins for the assembly of a sex pilus for the attachment, retraction, and recruitment of recipient cells. Following pilus assembly and recipient recruitment, successful processing and trafficking of mobile DNA elements transfer encoded helicases, enzymes which unwind DNA and facilitate the nicking, replication, and trafficking of transfer DNA to the internal side of the conjugation machinery. Transfer strand DNA is then inserted and then transferred using ATP-dependent transferases to the recipient bacteria where it must be circularized, genes expressed, and mobile element replicated (Willetts and Wilkins, 1984; Johnson and Grossman, 2015).

### Integrative Conjugative Elements

Integrative conjugative elements (ICE) are a unique form of insertable DNA vector that can be excised from the chromosome and transferred through conjugative sex between bacterial strains. ICE contain the genetic information that completely encodes for the machinery to enable its transmission. Unlike plasmids, ICE do not normally reside in the host cell as extrachromosomal genetic information. Instead, ICE are inserted sequences in the host chromosome and replicates as a passive element within the chromosome. Integration of ICE elements into target DNA sequences is dependent on the presence of sequence specific integration attachment (*att*) sites such as *attB* and a correlating recombination module encoded *att* site, such as *attP* (Wozniak and Waldor, 2010; Bauelos-Vazquez, 2017). Sequence binding between the *attP* and *attB* site by ICE vectors results in recombination and integration into the target DNA. This process results in duplicate flanking *att* sites on either end of the insertion increasing the total number of ATT sites in the target DNA sequence (Johnson and Grossman, 2015; Delavat et al., 2017). The prevalence of *att* sites varies in bacterial genomes; however, it is clear that ICE and associated *att* sites are widespread in bacteria so the specificity of ICE transfer and integration into bacterial hosts of varying backgrounds is not entirely clear (Cury et al., 2017; Guédon et al., 2017). Recently, examples of ICE that employ a site-independent integration using a separate DDE recombinases have been identified which has further added ambiguity to the host range of ICE vectors (Johnson and Grossman, 2015).

The mechanisms of ICE replication and transfer *in vivo* are still not completely understood, such as the steps of excision, maintenance, circularization, conjugative transfer, and integration in the gut environment (Pan et al., 2019). Thus, the continued observations and experimental studies of ICE are significantly important in the understanding, and prevention of, the spread of ARG both in the animal gut.

ICEs are typically modular, with weakly defined beginnings and ends as compared to other genetic vectors. ICE sequences are typically flanked by elements resembling transposons and viruses which allow them to randomly acquire new gene regions through recombination and inexact excision (Wozniak and Waldor, 2010). Davies et al. describe a novel ICE, ICEde3396 isolated from *Streptococcus dysgalactiae* (Davies et al., 2009). They determined through human isolate screening, that multiple *Streptococcus* isolates of group A, B, and G, possessed regions of the ICE, and some even contained all regions of the ICE. The authors then demonstrated by labeling the element with kanamycin resistance and *in vitro* mating that the element is conjugatively transferred from bacteria to bacteria, carrying the AR gene. They hypothesize that this integrative element can acquire DNA from each bacterial host that acquires it, and function as a vehicle for the dissemination of this information. While this was the observation of human isolates, it is not clear if this is true *in vivo* as it remains to be observed in the host.

There is anecdotal evidence that ICE are acting as vectors for modular evolutions where the dissemination of genetic information is facilitating the rapid adaption of bacterial strains to new metals and antibiotic substances. Davies et al. hypothesize that since β-hemolytic Streptococci have not developed resistance to penicillin, which is extensively used, by natural mutation, mobile genetic elements (MGE), such as ICE, are going to be the source of penicillin resistance acquisition in these bacteria (Davies and Davies, 2010). In *Enterobacteriaceae*, a prevalent family of bacteria implicated in the emergence and spread of antimicrobial resistance, ICE are highly prevalent such as in the pathogenicity islands of *Salmonella* with which also occurs in a large portion of non-*Salmonella Enterobacteriaceae* (Seth-Smith et al., 2012). Observational studies have shown the prevalence of MGE, including ICE, in bacterial strains isolated from populations of the human gut microbiotas, such as Actinobacteria, Bacteroidetes, Firmicutes, Proteobacterium, and Verrucomicrobium (Jiang et al., 2019). Furthermore, Coyne et al. reported evidence of extensive ICE transfer in the gut of humans. This study was observatory in nature and requires further experimental validation (Coyne et al., 2014).

In both human and animal host, *in vivo* examples of ICE gene transfer are almost exclusively observational. It is imperative to determine the *in vivo* roles of ICE experimentally. While reviews on the mechanisms and biology of ICE have been performed, a separate, detailed review of the *in vivo* models to study the transfer of ARG and VGs is desirable (Johnson and Grossman, 2015; Delavat et al., 2017; Botelho and Schulenburg, 2021), but outside the scope of this review.

### Conjugative Plasmids

Conjugative plasmids are extrachromosomal circular DNA molecules replicated by the host during cellular division and periods of bacterial conjugation (Couturier et al., 1988; Grohmann et al., 2003; Smillie et al., 2010; Arutyunov and Frost, 2013). They can be mobile, mobilizable, or non-mobile in nature. Mobile plasmids, which encode all genes for replication, transfer initiation, and transfer machinery, and mobilizable plasmids which encode the genes for replication and transfer initiation but not transfer machinery, are potent vectors for the exchange and transfer of genetic information. These genetic vectors often encode for virulence factors such as secretion systems, siderophores, and metabolic pathways, as well as ARG (Gibson et al., 1979; Goldstein et al., 1983; Wang et al., 2003; Diaz et al., 2012; Benzerara et al., 2017; Jamborova et al., 2017; Cabanel et al., 2018; Dolejska and Papagiannitsis, 2018). Recently, both mobile and mobilizable plasmids have been areas of major focus in the prevention of the emergence and spread of ARG in the context of agricultural and clinical environments (Marshall et al., 1981; Hoffmann et al., 1998; Lacey et al., 2017). While much research has been conducted on the mechanisms, rates, and inhibition of plasmid conjugation between bacteria in environments such as soil and wastewater treatment, there has been sparse research conducted on these topics in the context of the animal host.

### Bacterial Plasmid Conjugation and the Human Gut

Although direct experimentation in humans is difficult due to the ethics related to this nature of research, many observational studies have been performed that demonstrate the capability of the human microbiota to be involved in the plasmid-mediated HGT of AR and VG. The presence of plasmid-mediated AR and VG in clinically isolated samples is well documented (Corliss et al., 1981; Balis et al., 1996; Oppegaard et al., 2001; Winokur et al., 2001; Salyers et al., 2004; Hong et al., 2009; Aguado-Urda et al., 2012; Diaz et al., 2012; Liu et al., 2012; McInnes et al., 2020). These plasmid-mediated ARG and VGs have been shown, anecdotally, to be involved in plasmid conjugation and the emergence of novel resistant bacterial strains in human healthcare (Balis et al., 1996; Guiyoule et al., 2001; Winokur et al., 2001; Ohana et al., 2005; Daini and Akano, 2009; Goldfarb et al., 2009; Mahmood et al., 2014; Schweizer et al., 2019). However, due to limitations in human microbial research related to the introduction of antimicrobial resistance plasmids into living hosts, there has been a limit to the understandings available surrounding the factors that participate in regulating the process of plasmid-mediated HGT in and on humans. Many studies have postulated hypothetical host-microbial interactions (HMI) involved in regulating the process of plasmid-mediated HGT in the animal microbiota, but few of these HMI have been experimentally demonstrated in an *in vivo* environment (Macuch et al., 1967; Dunkley et al., 2007; Ziv et al., 2013; Gresham and Hong, 2015; Card et al., 2017; Li et al., 2020).

Many of these studies, understandably, target the gut as the specific microbiota niche to study (Macuch et al., 1967; Gevers et al., 2003; Dunkley et al., 2007; Card et al., 2017; Oladeinde et al., 2019). While many surfaces in and on the human body are believed to maintain resident niche microbiotas, the gut significantly outweighs the others in importance, abundance, and effect on host physiology and biology (Huddleston, 2014; Zeng and Lin, 2017; Gould et al., 2018). To approximate this host environment, researchers in the fields of microbial evolution, emergent AR, and bacterial-host interactions have proposed both *in vitro* and *in vivo* models for the human gut and methods to use these models to study the role of the bacterial host interaction on HGT. Additionally, while integrative and conjugative elements and natural transformation with free DNA may play a role in the transfer of genes in the gut, the use of specific models to study these are not well developed and the contribution they make to the emergence of antimicrobial resistance and virulence spread is not clearly understood. As such, this review will focus on bacterial plasmid conjugation as the primary vector for horizontal gene transfer and describe the models used for study.

## *In silico* Models

Bacterial plasmid conjugation is inherently complex and relies on the interplay of a multitude of factors including both DNA and protein regulation, as well as intra and extracellular signaling and interactions (Willetts and Wilkins, 1984; Cabezón et al., 2014; Bragagnolo et al., 2020). Determining the significance of each individual factor in the incidence and rate of conjugation in complex environments is thus inherently difficult. However, the use of mathematical models to approximate conjugation in these environments, and to help elucidate the role and significance of these factors in conjugation has offered a potential way to unravel this complex interaction. For example, the roles of bacterial growth, self-regulatory elements, and mating pair stabilization proteins have been integrated using computational models. However, these models were experimentally informed by *in vitro* studies and do not currently incorporate all the confounding factors that potentially regulate plasmid conjugation in complex host associated environments such as in the animal gut (Haft et al., 2009; Merkey et al., 2011; Campos et al., 2020).

While *in vivo* experiments directly in the human gut are not feasible, mathematical, and computational models for conjugation do give us insight into the prevalence and impact of bacterial conjugation in these complex environments (Tepekule et al., 2019; Li et al., 2020; Techitnutsarut and Chamchod, 2021). Recently, Tepekule et al. described the impact of host antibiotic treatment history on plasmid-mediated resistance evolution in human gut microbiota by utilizing a composite model of resistance evolution and microbiota dynamics (Tepekule et al., 2019). Their model consisted of a set of ordinal differential equations that represent the growth and change in distribution of microbial phylums over time correlated with the incidence of antimicrobial treatment and recovery periods. Using this mathematical model to simulate treatment histories based on published microbiome data, they determined three crucial factors regulating HGT significance in the gut of antimicrobial-treated individuals. The first factor was total days of drug exposure, or the time that the patient was administered antimicrobial drugs during treatment. Secondly, the duration of the drug-free period after the last treatment, or the length of time for recovery of the gut microbiota. Finally, the center of mass of the treatment pattern, or the abundance and distribution of treatment.

The role or impact of antibiotics in bacterial plasmid transfer in the complex gut microbiota has been further modeled *in silico* by Klümper et al. whom used both direct experimentation in pig fecal extract experiments as well as in a mathematical model informed by results from the extract experiments and incorporated parameter vales for both susceptible and resistant populations, as well as for the microbial community (Klümper et al., 2019). This study demonstrated a time-discrete mathematical model to approximate the transfer of antimicrobial resistance plasmids in a complex microbiota environment compared to *in vitro* broth conditions. Using this model, the authors postulate two mechanisms for reduction in the minimum selective concentration, including the relative increased cost of resistance, and the protective effect of the microbiota community on the susceptible phenotype.

These *in silico* models have been beneficial in postulating novel mechanisms involved in the rate and incidence of plasmid transfer; however, mathematical modeling is burdened with inherent limitations due to the nature of mathematical modeling itself (Sørensen et al., 2005; Bakkeren et al., 2019). This approach is dependent on the quality and abundance of available source data; and is limited by the availability of metadata, or associated information about the data; coverage, or completeness of the data and the metadata; and diversity of the source data, or the number of real-world scenarios covered by the data.

In the case of bacterial conjugation in the human gut, many knowledge gaps may provide substantial variance that support or contradict mathematical modeling results when the observed trends are applied to real-world gut plasmid transfer. These factors need to be experimentally determined and characterized using the available *in vitro* and *in vivo* models available before they can be incorporated into *in silico* models. After which, a more refined and accurate model may be producible that would more accurately reflect real-world plasmid transfer in the gut. These models do, however, provide important preliminary insight and are helpful in postulating new research questions for further experimental exploration in both *in vitro* and *in vivo* models of the gut.

Furthermore, mathematical and *in silico* modeling of bacterial conjugation in the environment, such as in biofilms, wastewater, and *in vitro* laboratory conditions, however, modeling of conjugation in the *in vivo* environment of the animal gut is still limited, largely due to the lack of these priory data required for the construction of such models (Haft et al., 2009; Merkey et al., 2011; Campos et al., 2020; Sutradhar et al., 2021). As such, more effort to define and implement *in vivo* models to identify and characterize important *in vivo* factors is required before significant conclusions are achievable with *in silico* modeling.

## *In vitro* Models

A significant task for *in vitro* studies is to identify environmental conditions measurable *in situ* that can approximate the conditions of the gut. The two most straightforward and common methods for *in vitro* conjugation assays are broth and solid surface conjugations. Broth conjugations include the growth of, and combination of, a donor and recipient strain at even or varying ratios (e.g., 1:1, 1,000:1, 1:1000, etc.) in either rich or defined media for a predetermined period (Fernandez-Lopez et al., 2005; García-Cazorla et al., 2018; Khajanchi et al., 2019; Ott et al., 2020; Jochum et al., 2021; Redweik et al., 2021). Further selective and differential plating of samples occurs for the selection and identification of donor, recipient, and transconjugant populations. Solid surface conjugation, likewise, involves the growth of donor and recipient strains in rich or defined media, these cultures are then placed onto the surface of either sterile agar or sterile filter paper applied to the surface of agar (Brown, 2016; Khajanchi et al., 2019). After a period, the cells are either scraped off the agar, or the filter paper is removed and vortexed in sterile buffer to generate a suspension. The resulting suspension is serially diluted and plated as in broth conjugations to enumerate each bacterial population of interest.

Both broth and surface methods work to approximate different conjugation environments in the context of the gut. Broth conjugations simulate the luminal conjugation between bacteria suspended in the heterogeneous matrix of intestinal material; while solid surface conjugation resembles, instead, the mucosal and epithelial niche environments where bacteria are stationary and anchored to a surface (Khajanchi et al., 2019). Although, these two methods can approximate the location and relation of bacterial cells in the gut during conjugations, they do not inherently represent the physiochemical, biochemical, and mechanical conditions of the gut environment. Confounding factors, such as pH, salinity, antimicrobial peptide (AMP) concentrations, antibodies, the host microbiota, and any number of yet characterized host factors potentially play a significant role in the efficacy of conjugations in the gut. The understanding of the basic mechanism by which these factors may or may not affect conjugation are significant to understand the emergence of novel antimicrobial resistant strains in human and animal health.

To understand these confounding factors more completely, much work has gone into developing more extensive *in vitro* models to simulate the human or animal guts. Incorporating factors such as pH, oxygen saturation, and metabolites may increase the insight garnered by these studies.

### Chemostat Models

Chemostats are important *in vitro* models used by microbiologists to simulate the complex gut environment. They involve the automated, or manual, regulation of pH, osmolarity, nutrient availability, and homogenization of a bacterial suspension matrix in a built environment. These models are useful in approximating a limited set of gut factors and produce a limited approximation of the gut environment of a variety of hosts. However, chemostat models do not incorporate the entirety of the responsive host factors elicited by the microbiota in the gut environment, such as antimicrobial peptide secretion, reactive oxygen release, or even adaptive immune mediators such as IgA antibodies. Additionally, chemostats generally assume homogeneity in the systems they model, which is not the case in the gut environments where different sections of the gut have differing physiological characteristics.

This was assessed somewhat by Card et al. who used a six-stage fermentative chemostat to model the chicken gut environment. In their study, they describe the use of six individual 20 ml fermentation vessels that were maintained for pH, temperature, atmosphere, and matrix homogenization that represented separate segments of the gut (Card et al., 2017). Using the chemostat model, the authors were able to show the transfer of a multidrug resistance plasmid from *Salmonella* to commensal *E. coli* strains found within the chicken fecal microbiota. This study demonstrated the transfer of a multidrug resistance plasmid from a common gastrointestinal contaminant to the microbiota of chickens in a simulated chicken gut *in vitro*.

Human chemostat models are also in use to determine the role of human gut microbiota and host factors in the incidence and rate of conjugative plasmid transfer. Rooney et al. used a triple stage chemostat model of the human gut to demonstrate the transfer of carbapenem resistance (CRE) genes from *Klebsiella pneumoniae* to the microbiota of CRE-negative human feces (Rooney et al., 2019). The three vessels, V1 (proximal small intestine), V2 (distal small intestine), and V3 (large intestine), were controlled for pH and alkalinity. Additionally, V1 was supplemented with growth media to maintain nutrient availability. All three vessels were stirred for homogeneity and sparged with oxygen free nitrogen gas to maintain anaerobic conditions. This study demonstrated the colonization, clonal expansion, and transfer of CRE genes within the human gut microbiota.

However, chemostat models, such as those in Card et al. and Rooney et al. employ total vessel homogenization and fail to fully replicate the local segregation of physiochemical conditions found in each individual region of the gut compartment that each vessel represents. This is important because the intraluminal conditions of the gut contents are often significantly different than those found in the mucosal lining of the epithelia, and these differences result in distinct microbial communities between the two regains (Donaldson et al., 2015). Factors such as pH gradients, secretory proteins, and nutrient bioavailability vary between the epithelial surface and the luminal content, and this has been shown to significantly impact the overall growth and activity of gut microbes such as *Escherichia coli* (*E. coli*) and other *Enterobacteriaceae* that are important in the emergence and spread of antimicrobial resistance through bacterial conjugation indicating that the use of these models may mislead or provide an inaccurate representation of what may occur in the complex gut environment (Poulsen et al., 1995; Licht et al., 1999). Licht et al. demonstrated drastic differences between a homogeneous chemostat model and *in vivo* transfer of plasmids in a streptomycin treated mouse model (Licht et al., 1999). Instead, a fixed continuous flow biofilm culture better represented the results observed in the murine model but offered limitations such as cessation of plasmid transfer after an initial period of high incidence.

Further studies on the use of chemostats are warranted, as they provide meaningful insight into potential roles of various physiochemical factors identified in the gut environment; however, their results should be interpreted with an objective understanding of the limitation of this model to approximate the gut environment. Future studied implementing chemostats should further include the addition of either specific or combinations of additional host factors to hopefully increase the accuracy and impact of the results obtained from their use such as mechanisms to generate static regions of low homogeneity that may be able to rescue some characteristics of the epithelial and mucosal conditions found *in vivo*. Additionally, supplemental addition of individual factors such as purified antimicrobial peptides or isolated host immunoglobulin could help elucidate the specific role of those factors in conjugation in a less complex approximations of the gut environments.

## *Ex vivo* Models

Limitations associated with simple *in vitro* studies, such as the lack of confounding host factors, has led to the need to identify and develop intermediate conditions that retain the ease of use and simplicity of traditional *in vitro* assays, but rescue a portion of complexity associated with host environments. To address this, a common practice in host-microbial interactions is to use host tissue *ex vivo*, or outside of the natural animal host (Bermudez-Brito et al., 2013; Machado and Sommer, 2014; Barrila et al., 2018; Stromberg et al., 2020). This approach offers advantages over traditional *in vitro* studies, such as maintaining a controlled environment while reincorporating the complex interactions associated with host tissues (Bermudez-Brito et al., 2013; Barrila et al., 2018).

### Cell Culture Model

While studying plasmid transfer in the context of the *in vivo* human gastrointestinal tract is not feasible, the use of human- and animal-derived gastrointestinal cell lines does give us a glimpse at the host biology involved in host-microbial interactions. Machado et al. describe the use of the human gastrointestinal carcinoma cell line, Caco-2, as an *in vitro* model of the human gastrointestinal system (Machado and Sommer, 2014). The authors grew these immortal intestinal cells on the surfaces of sterile transwell membrane inserts allowing for the development of a differentiated cell monolayer and the separation of media to the basal and the apical sides, approximating the separation of space in the human epithelial layer. After adding bacterial donors and recipients to the apical media in co-culture, the authors showed conjugation. Furthermore, they showed the reduction in bacterial conjugation in the presence of the Caco-2 cells when compared to broth conjugation. Using this model, the authors demonstrated an unidentified protein factor that modulates bacterial conjugation in *in vitro* cell line co-culture. While the authors have not yet further characterized the factor that is regulating conjugation in the gut, this study shows the importance of human cell culture in providing an insight into the host-derived factors that regulate conjugation in the gut.

The cell culture model, inherently, does not incorporate confounding factors of the human host, such as mucosal barrier, innate and adaptive immune function, or further factors important in regulation of bacterial metabolic pathways or inter species interactions such as those observed in the microbial fermentation of undigestible fibers into short chain fatty acids. However, many of these factors can be studied in more detail using this model by supplementing specific factors during conjugation. Cell culture serves as an important method for the future study of bacterial host interactions important in the regulation of bacterial conjugation and the emergence of novel antimicrobial resistant and virulent strains.

While cell culture provides an interesting model for the intestinal environment in conjugation events, it does not accurately represent healthy gastrointestinal tissue topology, biochemistry, nor diversity. Cell lines, such as Caco-2 cells, grow into monolayers and differentiate into complex cell surface topology; however, cell lines are typically cancerous or otherwise immortalized resulting in altered gene expressions and cellular response to stimuli and may vary even between cell lines of similar sources, e.g., Caco-2 and HT29 (Verhoeckx et al., 2015). As such, the use of healthy tissue is desirable. Explant tissue culture is currently used in numerous bacterial associations, pathology, and host response studies (Sunkara et al., 2011; Stromberg et al., 2018). However, there have not yet been any detailed studies using them as a model to demonstrate conjugation in association with healthy animal tissue *in vitro*. Studies in our laboratory are currently exploring this model to identify host responses in controlled *in vitro* assays (Ott et al., 2021).

## *In vivo* Models

While *in vitro* models provide a glimpse into the role of individual factors of the gut environment in bacterial conjugation and a useful insight into the mechanism that may be involved, a wholistic model is required to better understand the real-world role of host and microbial factors on bacterial conjugation (Ott et al., 2020). Animal models that offer both a reduced gut complexity and express conserved functions from the animal gut have recently been used to model bacterial conjugation *in vivo* (Stecher et al., 2012; Ott et al., 2020, 2021). While they are not entirely representative of the natural gut environment of humans, they do incorporate compounding factors such as host immunity, diet, genetics, and microbiota; that are not well accounted for in *in vitro* and *in silico* models (Petridis et al., 2006; Stecher et al., 2012; Ott et al., 2020, 2021).

### Arthropod Models

Arthropods are the most diverse and abundant group of animals on earth (Aguinaldo et al., 1997; Thomas et al., 2020). With significant co-occurrence in the environment with microbial communities, interaction between the two is a constant. The horizontal transfer of bacterial genetic information to arthropod hosts has previously been studied as a mechanism for both eukaryotic and prokaryotic evolution (Wybouw et al., 2016). However, the contribution of HGT between bacteria in the gut of arthropods resulting in the emergence of AR and virulent strains has not been extensively studied (Hoffmann et al., 1998; Fukuda et al., 2016, 2019). The mobility, and prevalence, of arthropod hosts in the environment, and specifically in the context of agricultural systems, make them an important reservoir for mobile AR and VG (Black et al., 2018; Fukuda et al., 2019). Furthermore, due to the dietary habits and lifecycles of many agricultural pests, exposure to populations of microbes sub-lethally exposed to antibiotics results in proliferate colonization of arthropod guts with bacterial strains harboring conjugatively transmissible elements, in the form of extrachromosomal plasmids (Hoffmann et al., 1998). As such, these complex gut-microbiota can function as reservoirs for the donation of these AR and virulence plasmids to pathogens and pathobionts resulting in the emergence of both human and agriculturally significant pathogens with novel phenotypes (Black et al., 2018; Fukuda et al., 2019).

The microarthropod *Folsomia candida* (Collembola), is important in this spread of mobilizable genetic material into microbial populations. Genetic information introduced into the gut microbiota of arthropod guts is further deposited by the host to soil and plant surfaces in agricultural environments (Hoffmann et al., 1998). This is significant if you consider the prevalence of HGT occurring on the surface of agricultural produce that participates in the colonization and spread of AR in the gut of humans (Maeusli et al., 2020). The arthropod endosymbionts of the genus *Rickettsia* have been shown to contain chromosomal and plasmid-encoded conjugation genes in addition to theoretical VGs involved in pathogenicity in humans (Weinert et al., 2009). However, it is not yet clear if this inherent plasmid conjugation is involved in the emergence of newly pathogenic bacteria that passage through these arthropod hosts and terminate as infections in humans.

While the significance of bacterial conjugation in these arthropod hosts in a clinical context is not clear, agriculturally significant strains are participating in conjugation. Poole et al. describe the transfer of a large AR plasmid from *Salmonella enterica* Serovar Newport to a laboratory *E. coli* (JM109) within the gut of the lesser mealworm beetle, *Alphitobius diaperinus* (Poole and Crippen, 2009). In this study, the authors demonstrated the *in vivo* transfer of AR from a commercially significant *Salmonella* pathogen to a laboratory strain *E. coli* after oral inoculation and colonization of the mealworm beetle, commonly associated with poultry litter and other poultry environments. These mealworms and other insects found in poultry environments are considered an important source for multi-drug resistance bacteria (McAllister et al., 1994, 1996; Wybouw et al., 2016; Fukuda et al., 2019).

The prevalence of HGT in the gut of arthropods is concerning as a source of novel resistance and virulence strains. The human pathogen, *Yersinia pestis*, the causative agent of the plague, has recently been shown to participate in high frequency conjugation with gut commensal *E. coli* donors *in vivo* in the midgut of the rat fleas *Xenopsylla cheopis* (Hinnebusch et al., 2002). The authors demonstrated the role of the gut microbiota of the alternate host as a source of additional AR plasmids in the emergence of novel plague strains with clinical significance. The model used in this study approximated the natural environment, and the authors hypothesize that the results may be like what could be seen in the wild. However, this has yet to be experimentally demonstrated and should be studied further to determine the significance of arthropod host microbiota on the emergence of bacterial pathogens of human importance.

There have been few experimental studies yet that describe the bacterial host interactions that regulate the conjugation of plasmids and other MGEs in the gut of arthropod hosts. The common housefly *Musca domestica L*. was shown to facilitate the intestinal transfer of the pCF10 AR plasmid among *Enterococcus faecalis* (Akhtar et al., 2009). This model, however, has not yet been used to study in-depth any host-bacterial interactions that regulate the rate or presence of conjugation in the gut. Our laboratory has recently demonstrated the use of *Drosophila melanogaster* as a model organism for arthropods, humans, and other animals. Using this model, we hope to utilize the vast genetic toolbox and reduced gut complexity to identify specific mechanisms by which the host regulates bacterial conjugation. We have shown a potential role of plasmid Inc. type and host genetics on regulating conjugation in the gut (Ott et al., 2021). However, this model offers a significant amount of variability and requires significant increases in sample size due to the limited volume of the gut. We are further validating and optimizing this model for continued use.

### Rhabditidae Model

The free-living nematode *Caenorhabditis elegans* (*C. elegans*) is extensively used to study genetics and cellular development due to its extreme simplicity and genetic tractability. Furthermore, due to its rapid multiplication time and ease of culture, they offer themselves as a desirable model for host bacterial interaction studies. Portal-Celhay et al. demonstrated *C. elegans* as a model for conjugative transfer of the R27 IncH1 plasmid (Portal-Celhay et al., 2013). The authors proposed a role of both age and genotype on the rate of intestinal conjugation between *E. coli* hosts. They identified age as a driver for an increased rate of conjugation with significant increases in older hosts (days 1–3 versus 4–7). Furthermore, they demonstrated that host genotype is a predictor for both conjugation frequency and transconjugant populations size within the gut of *C. elegans*.

However, the methods used in Portal-Celhay et al. study (Portal-Celhay et al., 2013) do not entirely rule out environmental conjugation, as the nematodes were grown on bacterial mats on the surface of growth agar, and conjugants could form outside of the host and then be subsequently ingested by the host. The role of environmental conjugation may be significant in the increase in conjugation identified with age as the transit time in the gut and volume of defecation varies with the age of nematodes (Portal-Celhay et al., 2013). The methods used with this model must be further refined to ensure that conjugation is occurring within the gastrointestinal tract of the animal host and not entering the gut through digestion.

### Phasianidae Model

Avian sources are believed to be the largest contributors to foodborne illnesses (Economic Research Service, 2015). Additionally, Liu et al. recently showed that microbial contaminants on the surfaces of commercial poultry samples not only harbor AR but can be phylogenetically linked to urinary infections in humans (Liu et al., 2018). Acting as a significant host to foodborne illness, agricultural and healthcare industries are especially concerned with the formation of AR and the ability of such a resistance to being spread to other organisms or consumers (Smith, 1970; Teuber, 1999). The avian gastrointestinal tract has previously been approximated *in situ* and *in vitro* (Card et al., 2017). *In vitro* chemostat models have been described previously; however, these methods do not incorporate host factors such as avian immunology, dietary interactions, the mating pairs, or the role of the host microbiome (Card et al., 2017). As such, *in vivo* studies in the chicken gut are still desired to determine the holistic regulation of bacterial plasmid conjugation.

In 1970, Smith et al. stated that the transmission of an R factor-mediated AR occurred readily *in vitro*, and that many researchers previously used modified *in vivo* hosts, such as antibiotic knockdowns and gnotobiotic birds, to study plasmid transfer in a host, however, none studied the spread of AR in a non-modified normal microbiota host (Smith, 1970). As such, the authors conducted plasmid transfer between different strains of *E. coli* and *S. enterica sub*sp. *enterica* Serovar Typhimurium in chickens. They concluded that not only was the resistance being transferred to the new bacterial hosts, but the organisms were also invading the tissue and were detectable in the liver. We have previously demonstrated the ability of large plasmids conferring both AR and virulence genes to normal gut microbiota members (Mellata et al., 2010). This is significant, as it shows transconjugant bacteria with acquired AR is transferring to areas of poultry tissues not automatically discarded at harvesting, such as is the intestines, in addition to the ready ability of poultry pathogens to spread AR and virulence genes to commensal organisms’ (Oosterom et al., 1983; Mellata et al., 2010).

HGT in the agricultural environment has been considered a source of emergence and spread of AR; however, it is also as source of plasmid-mediated spread of virulence factors (Lacey et al., 2017). Identifying the mechanistic change in DNA associated with the emergence of virulent strains with medical significance to humans is important. While random mutations act as a source for the emergence of novel genes, in the commercial environment, the horizontal transfer of large plasmids encoding whole AR and VG are more concerning due to the rapid spread possible in a population. Lacey et al. demonstrated the prolific spread of virulence plasmids between strains of *Clostridium perfringens* within the gastrointestinal tract of chickens without selection under antibiotic pressure (Lacey et al., 2017). Furthermore, this cohort hypothesis that this conjugation-mediated HGT may play a significant role in pathogenesis and evolution on *Clostridium* sp. and this needs to be further studied for significance.

The role of host factors, such as DNA, RNA, secretory proteins, and other macromolecules, is not entirely clear; however, studies in chickens have begun to provide some introductory insight. We previously showed the implication of cecal small RNA (sRNA) in plasmid transfer in the gut (Redweik et al., 2021). In this study, we demonstrated that the concentration of cecal sRNA is affected by prophylactic treatments giving to chicks and is proportional to the incidence of large plasmids in *E. coli* isolates from the ceca; *in vitro* assays showed a sRNA dose-dependent increases in bacterial conjugation in response to addition of isolated ceca small RNA. Further experimentation is required to better elucidate how cecal RNA populations may regulate bacterial conjugation and the incidence of large plasmids in the gut microbiota.

### Leporidae Model

Rabbits provide a valuable experimental model analog for humans and other animals (Hershberger et al., 2000; Peng et al., 2015; Hirt et al., 2017). While rabbits have notable differences in physiology, such as being primarily fermentative in gastrointestinal digestion as compared to humans, they have been used extensively as a model for a plethora of human infections and diseases (Ericsson, 2019). Rabbits were, and continue to be, invaluable in the effort to make fundamental discoveries in immunity such as in the development of the rabies vaccine. The use of this model for human gut physiology has its limitations; however, rabbits may serve as a developed, more complex, intermediate between *in vitro* studies and human physiology.

For example, Hirt et al. used New Zealand White Rabbits in an endocarditis model of *E. faecalis*. Using this model, the authors showed stimulation of virulence and plasmid transfer of the sex pheromone plasmid pCF10 (Hirt et al., 2018). Through *in vivo* rabbit experiments and *in vitro* human and rabbit sera experiments, the authors showed that plasmid pCF10, which harbors the gene for aggregative substance molecules, not only increases vegetative mass of endocarditis but is important in the induction of plasmid transfer upon exposure to human and rabbit sera. This work shows that human and rabbit plasma function as an interference agent in the normal signaling of the sex pheromone and induce higher rates of plasmid transfer. While the authors do show this behavior *in vivo* in rabbits, they do not show that it is the case in humans *in vivo*. Rabbits are a valuable model for human diseases; however, it is still unclear if this is the same interaction that can be observed *in vivo* of human hosts. The significance of this work is evident when considering methods to prevent plasmid transfer. Defining an immune response to strains containing plasmids may not be sufficient to prevent HGT, in fact, it may exacerbate it by interfering with normal conjugation regulation.

### Muridae Models

Observing plasmid transfer through conjugation in the mammalian gut proves to be difficult due to the variability in host factors, such as diet, environment, age, genetics, and microbiota. For example, the murine model for humans is difficult to use in the study of HGT as they confer a natural resistance to colonization by *Enterobacteriaceae* members, such as *E. coli* and *Salmonella* (Stecher et al., 2012; Ott et al., 2020). Both of which are significant contributors to the emergence and spread of novel AR plasmids as well as integrative elements (Stecher et al., 2012; Redondo-Salvo et al., 2020). Recently, Lasaro et al. demonstrated the isolation and use of an *E. coli* isolate for the colonization and replication in the gut of a murine model. While this does offer opportunities to use this *E. coli* strain as a recipient in *in vivo* conjugation experiments, it does not address the limitation of using alternate *Enterobacteriaceae* donors of interest such as human gastrointestinal pathogens (Lasaro et al., 2014). It is not clear what specific metabolic characteristics enable the reliable colonization and persistence in the gut microbiota, but the authors postulate that ability to participate in anaerobiosis and the expression of stress response regulators ArcA, CpxR, and RcsB are strong contributors to colonization. Identification of these pathways may lead to a better developed murine model for studies on conjugation in the gut and this warrant further exploration.

Mice can be conditioned chemically to prevent this natural resistance to *Enterobacteriaceae* colonization. The primary and most used method for this is the use of broad-spectrum antibiotics, such as streptomycin, to knock down the resident gut microbiota. Reduced microbial diversity and abundance in the gut permits colonization and persistence of *Enterobacteriaceae*, such as *E. coli* and *Salmonella*, within the gastrointestinal tract of mice. Alternatively, germ-free mice can be used to prevent the use of antibiotics and any associated effects of the drugs on the hosts physiology; however, both methods rely on ablating the natural gut microbiota that has a demonstrated role as crucial in normal host physiology and gut homeostasis (Stecher and Hardt, 2008; Round and Mazmanian, 2009; Broderick et al., 2014; Kim et al., 2014; Sannino et al., 2018; Lyte et al., 2019; Liao et al., 2020; Neil et al., 2021). For example, the gastrointestinal tissue of germ-free mice demonstrates significantly altered physiology and inflammation status than conventionally colonized mice (Brand et al., 2015). As a result, these models have a plethora of alternate effects such as altered gut brain axis function, diarrhea, increased gastrointestinal inflammation, and reduced macronutrient absorption (Brand et al., 2015; Lyte et al., 2019). These factors may interfere with observation of these studies or introduce additional variables that may be unaccounted for.

As a result of these limitations of chemical and germ-free models, an intermediate of germ-free and conventional microbiota is desirable for the observation of host and microbial interactions under non-selective host conditions. A model that rescues normal host physiology while offering a much less complex microbiota and allowing for stable colonization and persistence of *Enterobacteriaceae* species. To solve this issue, many studies have turned to using gnotobiotic mice, such as the Altered Schadler Flora (ASF) mice models (Brand et al., 2015; Lyte et al., 2019; Ott et al., 2020). ASF mice contain a defined set of eight bacterial species that approximate the gut of a healthy mouse while limiting the diversity of the gut (Brand et al., 2015). This host was recently shown to be a good model for *Enterobacteriaceae* colonization and *E. coli* pathology (Stromberg et al., 2018). Furthermore, this model was recently used by our team to show successful *E. coli* colonization of the gut as well as elucidate host factors important for the regulation of HGT between a foodborne *Salmonella enterica* subsp. *enterica* Serovar Kentucky and *E. coli* (Stecher et al., 2012; Ott et al., 2020). This model is desirable due to the control over inherently complex host factors otherwise unaccounted for. ASF mice are isolated in sterile, flexible film barrier housing and fed irradiated standardized diets and subjected to regulated night day light cycles in a temperature-controlled room. The genetics of these animals can also be controlled using inbred mouse lines.

The use of the ASF mouse model is continuing to be explored as a controlled model to determine the effects of any number of host factors on conjugation in the gut. It may be used to further identify the role of host immunity and novel vaccine targets on conjugation, as well as other immune mediators such as antimicrobial peptides. It can also be used to determine the role of diet or the environment on the stability and efficiency of HGT in controlled settings, unlike the case with many other models where variability in starting hosts leads to uncertainty in experimental results.

Sub-lethal antibiotic exposure (SLAE) exhibits effects on bacteria beyond cell damage and death. SLAE initiates alternate gene expression in exposed cells, such as initiating the stress response pathways, reducing metabolic activity, and stimulating conjugative plasmid transfer (Beaber et al., 2004). Furthermore, antibiotic treatments affect the gut microbiota of mammals by significantly changing the abundance and the distribution of a complex network of microbes (Midtvedt et al., 1986; Pérez-Cobas et al., 2013; Modi et al., 2014; Tormo-Badia et al., 2014; Candon et al., 2015; Theriot et al., 2016; Yang et al., 2018). SLAE results in the change of gut homeostasis by, changing both host and bacterial metabolites, alterations in microbial signaling, antimicrobial peptide expression, and immunoregulation, disruption of gastrointestinal cell regulation, and systemic dysregulation of host immunity (Zhang et al., 2019).

SLAE results in an increased conjugative transfer of plasmids of the critically important extended-spectrum beta-lactamases (ESBL) and other AR (Liu et al., 2019). This may be due to the selection for, and proliferation of transconjugant and AR strains in the absence of competition resulting in the increase in densities of the donor strain, or it may also be the result of the clearance of the microbial community and reseeding of the gut with donor strains from the population of resistant persister cells; this is not yet clear and needs to be explicitly studied to determine the significance of either pathway in these events (Bakkeren et al., 2019). However, either methods would be significant due to the current prevalence of antibiotic use and misuse in non-susceptible infections.

A primary knowledge gap in bacterial conjugation is the role of bacterial strain differences and plasmid-encoded genes and the effect of these factors on conjugation *in vivo*. Few studies so far have characterized the role of the plasmid Inc. type and associated gene sequences with the regulation of conjugation in the gut of mammals. While this has been studied in the context of the natural and built environments, soil, and wastewater, respectively; it has yet to be characterized in depth using animal models (Klümper et al., 2015). It was recently demonstrated that ESBL-plasmid positive *E. coli* strains of differing Inc. groups conferred a variation in conjugation in a streptomycin treated murine gut model (Benz et al., 2020). These changes in conjugation efficiency were attributed to the presence of various *tra* genes, and associated proteins, involved in the structure and function of the conjugation machinery on the plasmids. Furthermore, the authors of this study also demonstrated the role of the native plasmids hosts in modulating conjugation efficiency by comparing native plasmids hosts with a transconjugant host plasmid in competition experiments.

Supporting this notion of plasmid-encoded factors that regulate conjugation, Neail et al. reported highly efficient transfer of an IncI2 plasmid in the gut of a streptomycin treated murine model (Neil et al., 2020). The authors isolated and characterized 13 plasmids from enteric bacteria and used a common modified *E. coli* Nissle donor to test incidence and rate of conjugation in the gut of the antibiotic treated murine host. They identified that the IncI2 plasmid TP114 demonstrated significantly greater levels of conjugation, with almost 100% conjugation efficiency, measured as the proportion of recipient population which receives the plasmid. The authors further postulate that the presence of a specific T4SS pilus present in the I-complex plays a crucial role in conjugation observed in the gut, and is likely crucial to mating pair stabilization.

It is not yet clear if these factors are consistent with plasmids of other classes as the authors only examined those of IncF and IncI subsets. Furthermore, the donor and recipient strains used in this study were primarily isolated from human clinical samples. While this does give some insight into the role of clinical strains in direct human health, it is not clear if this accurately represents environmental isolates that may be the source of these clinically relevant multidrug resistance plasmids. The authors do clarify that a larger study with a different experimental design would need to be conducted to determine if other host strain factors, such as restriction-modification, CIRSPR-Cas, or other systems, are essential in the variation in the conjugation efficiency seen between the various donor and the recipient strains (Benz et al., 2020).

Variations in host genetics play a significant role in the microbial populations in the gut, as well as the interactions of these microbes and the host. We showed that when compounding factors such as diet, environment, and microbiota are controlled, variations in host genetics significantly contribute to the levels of conjugation in the gut of a murine model (Ott et al., 2020). So far, the genetic host factors responsible for variation between host genetics have not yet been elucidated but may be the result in variations in; gut immunity (antimicrobial peptides, antibody excretion, etc.), gut metabolite availability, and microRNA regulation and microbe-host signaling. Further studies will be required to determine the important host genetic factors involved in the regulation of the gut-mediated HGT between bacteria.

Host inflammation results in significant immune regulation of gut microbes; however, bacterial species such as those of the *Enterobacteriaceae* family can persist, bloom, and elicit continued inflammation in the guts of mammalian hosts (Lupp et al., 2007). This increased Enterobacterial density was shown by Stecher et al. to increase plasmid transfer by conjugation based on an increase in donor/recipient density in a detergent-induced inflammation murine mouse model (Stecher et al., 2012). However, this observation was not found to be consistent with our study when examined using an *IL-10* knockout chronic inflammation murine model compared to wildtype mice (Ott et al., 2020). Both studies used different donor and recipient strains so this relationship with host inflammation and conjugative plasmid transfer may be strain specific, or other compounding factors between model systems may not yet be known.

Future studies in mice of multiple inflammation types, as well as a greater number of donor and recipient strains, is needed to determine if this discrepancy between conjugation is strain-dependent or inflammation model-dependent interaction. The manifestation of inflammation between models can be mediated by different pathways and mechanisms and may be dependent on host genetics. For example, *IL-10* genetic knockout mice produce a chronic inflammation when exposed to bacterial stimuli, while the model in Stecher et al. was dependent on the use of CD8+ T cell injections targeting the hemagglutinin (HA) protein expressed on transgenic VILLIN-HA mice. These forms of inflammation differ in severity and pathology (Stecher et al., 2012; Ott et al., 2020).

In addition to mice, Rats have been used to demonstrate the role of dietary organism containing resistance plasmids in HGT in the gut. Jacobson et al. demonstrated the transfer of both *tet*(M) and *erm*(B) resistance plasmids from a food associated *Lactobacillus plantarum* to the pathogenic *E. faecalis* JH2-2 strain in the gut of gnotobiotic rats (Jacobsen et al., 2007). Furthermore, rats have been used to demonstrate both the absence and presence of transfer of dietary DNA in between the host diet and the gut microbiota (Kosieradzka et al., 2010; Nordgård et al., 2012).

## Conclusion

The study of HGT in human and animal guts is of dire importance to combat the emergence and spread of mobile ARG and VG plasmids. However, due to limitations in the study of the human microbiota, alternate models are important to determine experimentally how host and bacterial factor interactions affect the occurrence of HGT in the gut. *In vitro* models have played a significant role in the controlled study of isolated factors in synthetic environments. These models have a specific role to fill; however, they do not allow for the study of HGT in complex environments like those found in the gut of animals. *In vitro* models do not incorporate the combination of microbial, and host secreted factors such as antimicrobial peptides, reactive oxygen species, immune mediates like IgA, or physiochemical factors such as pH and osmolarity. Thus *in vivo* models that approximate the human gut are desirable.

The ASF model accomplishes this feat by allowing for controlled incorporation of each of these host- and microbiota-derived factors. However, it has yet to be used to extensively study the varied factors involved in regulating HGT, with only a few mechanisms proposed thus far. Additional models with these qualities are desired, such as an arthropod model. An arthropod model of HGT in the gut would be beneficial due to the immense genetic toolbox available in arthropod models, such as *D. melanogaster*, as well as the significantly reduced gut complexity due to the absence of adaptive immunity. Using these models, we can better determine the specific role of host and bacterial factors on conjugation and HGT in complex gut environments. Doing so may aid in elucidating novel pathways and the mechanism to inhibit or prevent the emergence of AR and VGs in the human and animal guts.

## Author Contributions

LO and MM: conceptualization, writing—original draft preparation, writing—review and editing, visualization, and funding acquisition. MM: resources, supervision, and project administration. All authors contributed to the article and approved the submitted version.

## Funding

Funding sources for this review were from the United States Department of Agriculture, National Institute of Food and Agriculture project IOW05679 (LO) and USDA Hatch project IOW04202 (MM). Figure was created with BioRender.

## Conflict of Interest

The authors declare that the research was conducted in the absence of any commercial or financial relationships that could be construed as a potential conflict of interest.

## Publisher’s Note

All claims expressed in this article are solely those of the authors and do not necessarily represent those of their affiliated organizations, or those of the publisher, the editors and the reviewers. Any product that may be evaluated in this article, or claim that may be made by its manufacturer, is not guaranteed or endorsed by the publisher.
